# Coffee Pulp: A Natural Alternative for Control of Resistant Nematodes in Small Ruminants

**DOI:** 10.3390/pathogens12010124

**Published:** 2023-01-11

**Authors:** Gabino López-Rodríguez, Adrian Zaragoza-Bastida, David Emanuel Reyes-Guerrero, Agustín Olmedo-Juárez, Benjamín Valladares-Carranza, Luis Fernando Vega-Castillo, Nallely Rivero-Perez

**Affiliations:** 1 Instituto de Ciencias Agropecuarias, Área Académica de Medicina Veterinaria y Zootecnia, Universidad Autónoma del Estado de Hidalgo, Rancho Universitario Av. Universidad km 1. Ex-Hda. de Aquetzalpa, Tulancingo C.P. 43600, Hidalgo, Mexico; 2 Centro Nacional de Investigación Disciplinaria en Salud Animal e Inocuidad (CENID SAI-INIFAP), Carrtera Federal Cuernavaca-Cuautla No. 8534/Col. Progreso, Jiutepec C.P. 62550, Morelos, Mexico; 3 Centro de Investigación y Estudios Avanzados en Salud Animal, Facultad de Medicina Veterinaria y Zootecnia, Universidad Autónoma del Estado de México, km 15.5 Carretera Panamericana, Toluca-Atlacomulco, Toluca C.P. 50200, Estado de México, Mexico

**Keywords:** coffee pulp, eggs hatching inhibition, GIN, benzimidazoles resistance

## Abstract

Goat production in Mexico is an important economic activity that is affected by different gastrointestinal nematode (GIN) species. GINs resistant to commercial anthelmintics have been reported. Plant extracts or agro-industrial by-products, such as coffee pulp, have been proposed as control alternatives, given their secondary metabolite content. The aim of the present study was to determine the anthelmintic activity of the hydroalcoholic extract of coffee pulp against benzimidazole-resistant GINs. Stool samples were collected from goats, from which GIN eggs were identified and quantified. Molecular techniques confirmed the genus of GINs and their benzimidazole resistance profile. The percentage of egg hatching inhibition (% EHI) and larval mortality (% LM) with the hydroalcoholic extract of coffee pulp was determined at concentrations from 200 to 0.39 mg/mL. The genera *Haemonchus* spp. and *Trichostrongylus* spp. were identified, and the presence of the β-tubulin gene mutation, associated with benzimidazole (BZ) resistance, was determined. Hydroalcoholic extract of coffee pulp inhibited 100% of egg hatching at 200 and 100 mg/mL, with no larvicidal effect at the evaluated concentrations.

## 1. Introduction

Goats are bred in Mexico for the production of meat intended for sale and consumption, as well as the production of milk intended both for human consumption and the production of cheeses and typical sweets, with a production of more than 160,000 L per year [1]. Goat production systems in Mexico are heavily dependent on grazing on communal lands, and they contribute significantly to the livelihood of farmers [2]. The presence of parasites such as gastrointestinal nematodes (GINs) can reduce production by 10 to 40%, affecting feed intake and feed conversion ratio [3].

Sheep and goats are prone to parasitic infections because their reproduction and rearing are carried out collectively; nematodes of the Trichostrongylidae family are the most frequent parasites. According to Herrera et al. [4], mixed infections have been found to be common epizootiological events, with a variety of nematode species located in different portions of the digestive tract, the most common species being *Haemonchus* spp., *Cooperia* spp., *Bunostomum* spp., *Nematodirus* spp., *Trichostrongylus* spp., *Trichuris* spp., *Chabertia* spp., and *Oesophagostomum* spp.

In recent years, the control of helminthiasis has focused on treatment using mainly anthelmintics such as macrocyclic lactones, imidazothiazoles, and benzimidazoles. This chemical strategy has led to a rapid emergence of anthelmintic resistance in all livestock hosts due to the inappropriate use of a misguided strategy [5]. The development of variable degrees of drug resistance among nematodes has been reported for all groups of anthelmintics [6]. The prevalence of resistance has increased globally, and worryingly, a further increase can be expected due to massive drug administration, which creates selection pressure for resistance [7].

The availability of new anthelmintic therapy will be essential in the coming years, and considerable efforts must be made to develop new alternative anthelmintic control therapies, especially those with efficacy in nematodes resistant to classical anthelmintics [8]. Effective, sustainable methods and strategies have emerged with a sustainable environmental approach, such as the use of plant extracts or agro-industrial waste [9].

Some extracts of agro-industrial waste have been evaluated in recent years in order to find an application for products that end up being discarded. For instance, shea meal (residue from the nuts of *Vitellaria paradoxa*) has been demonstrated to be effective against the L3 of *Ascaris suum* [10]. Grape pomace and its extract showed activity against eggs and larvae of *Haemonchus contortus* (HC) in vitro [11].

In 2022, Castañeda-Ramírez et al. reported an in vitro anthelmintic effect against HC for ten extracts obtained from coffee pulp waste (*Coffea canephora*), corn comb waste (*Zea mays*), grass hay pangola (*Digitaria eriantha*) and different mixtures of these materials. They were more effective at inhibiting egg hatching than increasing larval mortality [12]. In 2016, Ortiz-Ocampo et al. reported an in vitro anthelmintic effect of *Coffea arabica* percolate on the exsheathment of HC. The revaluation of these agro-industrial residues, such as coffee pulp, is an emerging field with potential application in the production of small ruminants due to ecological and economic implications [13].

The aim of this study was to evaluate the anthelmintic activity of the hydroalcoholic extract of coffee pulp against resistant GINs isolated from goats.

## 2. Materials and Methods

### 2.1. Location 

One hundred and ten samples were collected randomly from 10 farms (n = 11) in the municipality of Tepeapulco, Hidalgo, Mexico, through stratified sampling. The geographical coordinates were 19°47′06″ north latitude and 99°33′11″ west longitude, with an altitude of 2508 m above sea level. The area has a temperate and temperate sub-humid semi-dry climate with rainfall in summer. The average monthly temperatures in the municipality range between 10.9 °C in December and January, which are the coldest of the year, to 16 °C in May and June, which record the highest temperatures. The objects of the study were goats, with an age range from 1 to 4 years under semi-intensive breeding systems with access to free water and grazing during the first 8 h of day and night confinement, with a history of deworming with derivatives of the benzimidazole family and macrocyclic lactones, without prior coproparasitological examinations. Animals were weighed for dosing.

### 2.2. Collection and Processing of Samples

Faecal samples (5–10 g) were collected directly from the rectums of the animals into the hermetic containers and processed for 12 h. The goats were managed following the care/welfare and non-unnecessary suffering standard regulations of NOM-051-ZOO-1995, NOM-062-ZOO-1999, and the Ley Federal de Sanidad Animal. The presence of nematode eggs was determined by the flotation technique, and the quantification of eggs per gram of faeces (EPG) was performed using the McMaster technique. The methodologies used were described by Hansen and Perry [14].

#### Obtaining Gastrointestinal Nematode (GIN) Infective Larvae

The GIN larvae were obtained from the mixed faecal material (10 g) from each farm. Faecal cultures were prepared by mixing faeces with polystyrene particles in Petri dishes and were incubated at room temperature (28 °C) for 17 d. After this period, the infective larvae (L3) were extracted from faecal material using the Baermann technique [14]. Then, the L3 was cleaned by density gradient (40% saccharose) and centrifugation and was kept at 4 °C until use.

### 2.3. Gastrointestinal Nematode Identification by Multiplex Polymerase Chain Reaction (PCR)

The genomic DNA (gDNA) was extracted via a commercial kit (Wizard^®^ Genomic DNA, Madison, USA) following the manufacturer’s protocol, with 10,000 L3 larvae washed and exsheathed with 3% sodium hypochlorite. The extracted gDNA was quantified in a nano-photometer (Implen, München, Germany) and was stored at −20 °C until use. The GINs were identified following the methodology proposed by Zarlenga et al. [15]. The forward (Fw) and reverse (Rv) primer sequences and the accession numbers are reported in Table 1. The amplified products were visualised in 3% agarose stained with ethidium bromide (Sigma-Aldrich, St Louis, MO, USA) using a UV photodocumenter (UVP, Upland, CA, USA).

### 2.4. Resistance/Susceptibility Assays through Allele-Specific Chain Reaction (AS-PCR) 

The diagnosis of resistance was made according to the methodology described by Mondragon-Ancelmo et al. [16]. The identification of the polymorphism responsible for benzimidazole resistance was conducted with allele-specific PCR, using the mutation in the β-tubulin gene residue 200. This method requires two or more oligonucleotide pairs flanking the fragment of interest for the identification of benzimidazole (BZ) resistance. The first was performed in a volume of 20 µL. Nuclease-free water (PROMEGA, San Luis Obispo, CA, USA), 10 ng gDNA, and 1 μL (20 μM) of the short sequences of nucleotides Pn1 (forward) and Pn2 (reverse) were used to obtain the first fragment (Table 2). The obtained amplification product was used as a DNA template for the second reaction with two different oligonucleotide pairs (P1, P2; P3, P4) to differentiate between resistant populations (250 bp), susceptible populations (550 bp), and/or heterozygotes. For all AS-PCR reaction mixtures, the amplification conditions were denaturation at 94 °C for 5 min, followed by 33 denaturation cycles at 94 °C for 55 s, annealing at 60 °C for 55 s, extension to 72 °C for 55 s, and a final extension to 72 °C for 10 min using a Touch Thermal Cycler C1000 (Bio Rad, Mexico City, Mexico). The amplified products were visualised in 3% agarose stained with ethidium bromide (Sigma-Aldrich, St Louis, MO, USA) using a UV photodocumenter (UVP, Upland, CA, USA).

### 2.5. Obtaining the Coffee Pulp Hydroalcoholic Extract 

The coffee pulp was collected in the Municipality of Huatusco, Veracruz de Ignacio-Llave (19°08′56″ N 96°57′58″ W). For identification of the plant, we consulted with the herbarium of the Autonomous University of Mexico (UNAM); the plant was verified to be *Coffea arabica* L. (IBUNAM: MEXU:1375798). In accordance with the procedure described by Rivero-Perez et al. [17], the dried vegetal material (500 g) was macerated with a hydroalcoholic solution (70% water: 30% methanol, 1.5 L) at 25 °C for 72 h. Subsequently, it was filtered with cotton and filter paper (Whatman^®^, Maidstone, UK). Later, the extract was concentrated using a rotary evaporator under reduced pressure (Büchi-R-300, Flawil, Switzerland). The extract was refrigerated (4 °C) until use.

### 2.6. Anthelmintic Activity of Coffee Pulp Hydroalcoholic Extract 

#### 2.6.1. Egg Hatching Inhibition (EHI) Test 

GIN eggs were obtained from a pool by mixing 10 g from each farm. For the EHI test, 50 g of faeces was washed in 200, 100, 75, and 37 µm sieves to recover and concentrate the nematode eggs, followed by density gradient centrifugation with saturated saline and rinsed with distilled water. The obtained clean egg solution was adjusted to a concentration of 150 eggs/50 µL.

The determination of anthelmintic activity was carried out according to the methodology described by Rivero-Perez et al. [17]. The EHI test was performed in 96-microtitration plates. Final concentrations of the hydroalcoholic extract of coffee pulp (200, 100, 50, 25, 12.5, 6.25, 3.12, 1.56, 0.78, and 0.39 mg/mL) were evaluated using distilled water as a negative control, and ivermectin at (5 mg/mL) as a positive control (Sigma-Aldrich, St. Louis, MO, USA), with four repeats each. An aqueous suspension of 50 µL containing the parasite eggs (150 ± 15) was pipetted into each well. Then, 50 µL aliquots of the treatment and controls were added, giving a total volume of 100 µL. The plates were incubated at a temperature of 28 °C for 48 h. After this period, the total number of eggs and larvae (L1 and L2) in each well was counted. The EHI percentage was estimated for each treatment group using the following formula:

(1) $$\%\;EHI=\frac{number\;of\;eggs}{number\;of\;larvae+number\;of\;eggs}*100$$

#### 2.6.2. Larval Mortality Test

This assay was performed in 96-well microtitration plates using GIN infective larvae (L3). Larval pellets (3000–5000 L3) were subjected to an ecdysis process induced with 3% sodium hypochlorite (Sigma-Aldrich, St. Louis, MO, USA) for 5 min at room temperature; the hypochlorite was removed with distilled water until a solution of clean larvae adjusted to 150 larvae/50 µL was obtained. The treatments were the hydroalcoholic extract (at 200, 100, 50, 25, and 12.5 mg/mL), distilled water as a negative control, and ivermectin (5 mg/mL) as a positive control. Fifty microliters of an aqueous suspension containing 150 ± 15 GIN larvae was deposited in each well. Then, 50 µL aliquots of extract and controls were individually added to each well. The plates were incubated at a temperature of 28 °C for 48 h. The criteria for estimating larval mortality included the count of live and dead larvae contained in aliquots of 10 μL (n = 10). The mortality percentage was estimated for each treatment group using the following formula:

(2) $$\%\;LM=\frac{number\;of\;live\;larvae}{number\;of\;dead\;larvae+number\;of\;total\;larvae}*100$$

### 2.7. Statistical Analysis 

The EHI and larval mortality percentages were normalised using a square root transformation and analysed based on a completely randomised design. The data obtained were analysed by analysis of variance (ANOVA) and a Tukey comparison of means at a confidence level of 95 %. The analysis of the information was performed using SAS software version 9.0 (SAS, Cary, NC, USA).

## 3. Results

### 3.1. Identification of GIN by Flotation Technique and Determination of Parasitic Load

**Figure 1:**
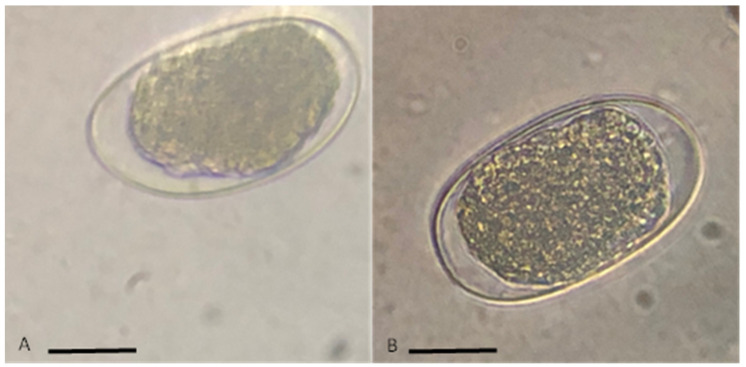
GIN eggs of Strongylida order observed under an optical microscope (100×): *Trichostrongylus* spp. (**A**) and *Haemonchus* spp. (**B**). Scale bar: 10 µm.

According to their morphometric characteristics, the GINs observed in the samples of the animals of the 10 farms were nematodes of the Strongylida order, *Haemonchus* spp. (Figure 1A) and *Trichostrongylus* spp. (Figure 1B).

Values for EPG showed that 30% (3 of 10) of farms presented a mild level of infestation (50–800), followed by 20% (2 of 10) with moderate infestation (800–1200) and 50% with a severe infestation (>1200) (Table 3).

### 3.2. Gastrointestinal Nematode Identification by Polymerase Chain Reaction (PCR)

The results of genotype testing showed amplification products of 243 and 176 bp, corresponding to the genera *Trichostrongylus* spp. and *Haemonchus* spp., respectively, in 10 sampled farms, with no amplifications of the remaining genera observed (Table 4). 

### 3.3. Resistance/Susceptibility Assays 

**Figure 2:**
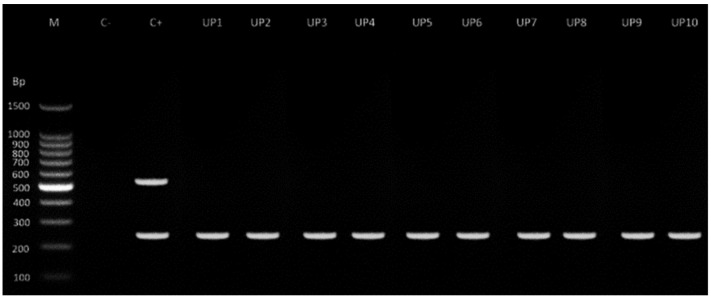
Representative gel PCR products. M, molecular marker; C-H_2_O, C+ heterozygous strain, UP farm.

The amplification products for the diagnosis of resistance (Figure 2) corresponded to 250 bp, associated with benzimidazole resistance in 10 farms, with no detection of susceptibility alleles (550 bp).

#### Determination of Anthelmintic Activity

The results of the anthelmintic activity assay (Table 5) showed that the hydroalcoholic extract of coffee pulp exhibits activity against egg-phase GINs, with 100% EHI observed with 200 and 100 mg/mL, showing statistical differences (*p* < 0.05) with ivermectin. A dose-dependent behaviour was observed; the lower the concentration of the hydroalcoholic extract of the coffee pulp, the lower the effect on egg-hatching inhibition. 

Regarding larval mortality (LM), the highest mortality recorded was 2.07%, with no statistically significant difference to the negative control (H_2_O); therefore, at the doses evaluated, the hydroalcoholic extract of coffee pulp showed no larvicide effect.

## 4. Discussion

Parasitosis caused by GINs in small ruminants is recognised as a disease of economic importance around the world. GINs cause a low productive yield and even the death of young animals. Thus, together with anthelmintic resistance, they represent a potential risk to current livestock.

The results obtained in the present study show that the goat farms in the municipality of Tepeapulco, Hidalgo, Mexico are semi-intensive; their characteristics (day grazing, night confinement, and forage as diet complement) and the absence of health-management strategies are the factors that determine the presence of pathogens such as parasites. According to Galaviz-Rodriguez et al. [18], the production systems that predominate in Mexico are semi-intensive and extensive. These farms rely on the use of vegetation in the region to reduce production costs, leaving animals exposed to constant contact with pathogens, especially during grazing hours.

On the other hand, the SADER [1] has identified that goat production in Mexico has an important role in the Comarca Lagunera and the Bajio regions, which are zones with dry or semi-dry climates to which goats adapt very well. The municipality of Tepeapulco is located in the central region of Mexico, where the dry or semi-dry climate is less extreme compared to the Bajio region; nevertheless, Tepeapulco has characteristics of a semi-dry climate that allows easy adaptation of goats to places where vegetation is scarce, allowing high sustainability under grazing conditions [19].

Unlike large commercial companies with highly technical and intensive production systems, where the productive capacity of animals is maximally used, and high yields are obtained [2], in semi-intensive production systems in which adequate sanitary and productive management are not considered, such as those in the present investigation, the incidence of diseases is high, while sanitary, productive, and reproductive parameters are low. In this kind of production system, the incidence of GINs is high, mainly in the spring and summer seasons due to rain, relative humidity, and temperature.

Morphological identification showed the presence of the GINs *Haemonchus* spp. and *Trichostrongylus* spp. with different levels of infestation—light (30%), moderate (20%), and heavy (50%). According to Suarez et al. [20], one disadvantage of sheep is their poor ability to regulate infection by helminths. In contrast, Alva-Perez et al. [21] reported that goats are more rustic and better regulate these infections. The infections do, however, cause a delay in their growth.

The level of infection is considered an indicator of sanitary management or the effectiveness of deworming programs and is used as a support tool to know the severity of the clinical or subclinical course of helminthiasis. These values, according to Hansen and Perry [14], change according to the number of adult parasites established in the gastrointestinal tract, the prolificacy of parasite species, and the level of immunity, age and physiological stage of the animals. These characteristics explain the variations in infection levels reported in the present investigation.

The presence of the genera *Haemonchus* spp. and *Trichostrongylus* spp. was confirmed by PCR. These genera have been reported previously by Ronquillo et al. [22] in the southeast of Puebla in grazing sheep, with *Haemonchus* spp. being the most prevalent, followed by *Cooperia* spp. and *Trichostrongylus* spp. The high prevalence of *Haemonchus* spp. and *Trichostrongylus* spp. has been associated with the high fertility rate of these genera, which increases the genetic diversification factor for the distribution of resistance alleles in the population [14].

Benzimidazole-resistant GINs have been reported in different regions of Mexico, with a high incidence of resistance in tropical regions. Torres-Acosta et al. [23] reported that this phenomenon has increased in recent years in the south-southeast region compared to the central region of Mexico due to climatic characteristics that increase the prevalence of GINs, the administration of anthelmintics and, thus, the selection of drug-tolerant individuals.

Previous research has reported resistance to anthelmintics in different zones of Mexico. For instance, Mondragón-Ancelmo [16] identified benzimidazole resistance alleles in *Trichostrongylus* spp. and *Cooperia* spp. isolated in sheep samples from the temperate zone of Mexico (Estado de México). Similar results were observed in the present study, where benzimidazole resistance alleles were identified in *Haemonchus* spp. and *Trichostrongylus* spp. in goats from Apan Valley (Hidalgo), Mexico.

There are many factors involved in anthelmintic resistance, such as the transfer of animals between farms, deworming in critical periods (winter), and the high density of animals. For many years, the administration of anthelmintic drugs was the main strategy used for the control and treatment of diseases of parasitic origin; however, the lack of criteria for the choice, dosage, and frequency of use of anthelmintic drugs, among other factors, has stimulated the generation of strains of parasites resistant to these pharmacological substances [16,24].

In response to the problem of anthelmintic resistance, the scientific community has focused on the search for functional, economic, innocuous and environmentally friendly alternatives for the control of the main parasites in small ruminants [9], such as products obtained from plants.

Evaluation of coffee pulp hydroalcoholic extract showed that concentrations of 200 and 100 mg/mL inhibited 100% of egg hatching of BZ-resistant GINs obtained from goats. In 2022, Castañeda-Ramírez et al. evaluated *Coffea canephora* pulp hydroalcoholic extract and did not find egg-hatching inhibition on a monospecific strain of HC resistant to ivermectin and BZ [12]. Zaragoza-Bastida et al. [25] evaluated *Cassia fistula* hydroalcoholic extract and reported 21 and 30% of IEH at 50 and 6.25 mg/mL, respectively. Better results were observed with *Coffea arabica* pulp hydroalcoholic extract, as reported in the present study.

On the other hand, for %LM, the hydroalcoholic extract of coffee pulp showed low activity. Castañeda-Ramírez et al. [12] calculated 10189.7 µg/mL as the effective concentration of hydroalcoholic extract of *Coffea canephora* to achieve 50% (EC_50_) larval mortality in ivermectin and BZ-resistant monospecific strain of *Haemonchus contortus*. In the present study, this concentration (EC_50_) was not calculated since the extract presented low activity, which could be due to the fact that the evaluation was carried out with a mixed strain of field NGI. In contrast, Zaragoza-Bastida et al. [25] reported mortality percentages of 29.65 to 25.67% with concentrations of 50 to 6.25 mg/mL against a mixed strain of field NGI, reporting better results than those obtained in the present study.

Hydroalcoholic extract of coffee pulp had better activity in terms of egg-hatching inhibition compared to larval mortality; this difference can be explained by the fact that L3 larvae are formed by more complex structures, coupled with different detoxification mechanisms that allow them to survive in more hostile environments compared to the egg stage [5]. Mkandawire et al. [26] reported that the structural composition of eggs is more sensitive and susceptible to damage by extrinsic factors that interfere with signalling, water, and sugar exchange or important enzymes in metabolism and development.

## 5. Conclusions

In the present study, the presence of the β-tubulin gene mutation, associated with resistance to benzimidazoles, was identified in the genera *Haemonchus* spp. and *Trichostrongylus* spp. from goats in Tepeapulco, Hidalgo, Mexico. The evaluation of the anthelmintic activity of the hydroalcoholic extract of coffee pulp allowed the determination that this extract inhibits the egg hatching of nematodes resistant to benzimidazoles. Hydroalcoholic extract of coffee pulp could be considered as an alternative for control of GINs resistant to benzimidazoles. However, more studies are required to determine the safety of this extract before performing a challenge in vivo.

## Figures and Tables

**Table 1:** Sequence of primers used for the molecular identification of each genus of gastrointestinal nematodes.

Nematode Genera	Bp	Sequence Fw ^1^ (5′-3′)	Sequence Rv ^2^ (5′-3′)
*Haemonchus* spp.	176	CATTTTCGTCTTGGGCGATAT	TGAGACCGCACGCGTTGATTCGAA
*Teladorsagia* spp.	257	GCAGAACCGTGACTATGGTC	GACAAGGAGATCACGACATCAGCAT
*Cooperia* spp.	151	TCGATGAAGAGTTTTCGGTGTTC	TTCACGCTCGCTCGTGACTTCA
*Oesophagostomum* spp.	329	CAGGGTCAGTGTCGAATGGTC ATTGTCAAATA	CAGGGTCAGTGGTTGCAATACAAATGATAATT
*Trichostrongylus* spp.	243	TAAAAGTCGTAACAAGGTATCTGTAGGT	GTCTCAAGCTCAACCATAACCAACCATTGG

^1^ Forward; ^2^ Reverse.

**Table 2:** Sequence of primers used for molecular diagnosis of benzimidazole resistance in gastrointestinal nematodes.

Primer	Sequence Fw ^1^ (5′-3′)	Primer	Sequence Rv ^2^ (5′-3′)
Pn1	5′GGCAAATATGTCCCACGTGC3′	Pn2	5′GAAGCGCGATACGCTTGAGC3′
P1	5′GGAACGATGGACTCCTTTCG3′	P2	5′ATACAGAGCTTCGTTGTCAATACAGA3′
P3	5′CTGGTAGAGAACACCGATGAAACATA3′	P4	5′GATCAGCATTCAGCTGTCCA3′

^1^ Forward; ^2^ Reverse.

**Table 3:** Level of infestation associated with the average egg per gram of faeces (EPG) for gastrointestinal nematodes.

Farm	EPG Average, Infestation Level		
	Light (50–800)	Moderate (800–1200)	Heavy (>1200)
1	600 ± 183		
2		850 ± 135	
3	500 ± 378		
4			1550 ± 287
5	350 ± 125		
6		600 ± 128	
7			2300 ± 209
8			2350 ± 266
9			1450 ± 226
10			1500 ± 303

EPG, mean ± standard deviation.

**Table 4:** Genotyping of gastrointestinal nematodes of goats by polymerase chain reaction (PCR).

	PCR Amplification Products (bp)				
Farm	329	257	243	176	151
1	Nd	Nd	+	+	Nd
2	Nd	Nd	+	+	Nd
3	Nd	Nd	+	+	Nd
4	Nd	Nd	+	+	Nd
5	Nd	Nd	+	+	Nd
6	Nd	Nd	+	+	Nd
7	Nd	Nd	+	+	Nd
8	Nd	Nd	+	+	Nd
9	Nd	Nd	+	+	Nd
10	Nd	Nd	+	+	Nd

+ amplification, Nd Not detected.

**Table 5:** Anthelmintic activity of hydroalcoholic extract of coffee pulp against GINs isolated from goats.

Treatments (mg/mL)	%IEH	% LM
Distilled water	1.43 ± 0.5 ^k^	3.2 ± 0.3 ^b^
Ivermectin (5 mg/mL)	95.33 ± 0.5 ^b^	99.46 ± 0.8 ^a^
EPC 200	100 ^a^	2.07 ± 0.1 ^bc^
EPC 100	100 ^a^	1.93 ± 0.3 ^bc^
EPC 50	92.87 ± 0.5 ^c^	1.77 ± 0.2 ^bc^
EPC 25	90.97 ± 0.1 ^d^	1.63 ± 0.3 ^bc^
EPC 12.5	88.78 ± 0.4 ^e^	0.61 ± 0.3 ^c^
EPC 6.25	67.87 ± 0.6 ^f^	0
EPC 3.125	49.58 ± 0.5 ^g^	0
EPC 1.56	31.40 ± 0.4 ^h^	0
EPC 0.78	16.90 ± 0.5 ^i^	0
EPC 0.39	7.63 ± 0.1 ^j^	0

Mean standard error and value of *p* ≤ 0.05. ^a–k^ Different letters within the same column represent statistically significant differences between treatments. EPC, hydroalcoholic extract of coffee pulp.

## Data Availability

Data are contained within the article.
